# Therapeutic potential of Pien Tze Huang in colitis-associated colorectal cancer: mechanistic insights from a mouse model

**DOI:** 10.1186/s12935-024-03428-9

**Published:** 2024-07-17

**Authors:** Liya Liu, Youqin Chen, Sijia Liu, Xinran Zhang, Liujing Cao, Yulun Wu, Yuying Han, Guosheng Lin, Lihui Wei, Yi Fang, Thomas J. Sferra, Anjum Jafri, Huixin Liu, Li Li, Aling Shen

**Affiliations:** 1 Affiliated Rehabilitation Hospital of Fujian University of Traditional Chinese Medicine, Fuzhou, Fujian China; 2 Fujian Key Laboratory of Integrative Medicine on Geriatrics, Fujian University of Traditional Chinese Medicine, Fuzhou, Fujian China; 3 Clinical Research Institute, The Second Affiliated Hospital & Academy of Integrative Medicine, Fujian University of Traditional Chinese Medicine, Fuzhou, Fujian China; 4 Fujian Collaborative Innovation Center for Integrative Medicine in Prevention and Treatment of Major Chronic Cardiovascular Diseases, Fuzhou, Fujian China; 5 Department of Pediatrics, Case Western Reserve University School of Medicine, Rainbow Babies and Children’s Hospital, Cleveland, OH USA; 6 Innovation and Transformation Center, Fujian University of Traditional Chinese Medicine, Fuzhou, Fujian China; 7 Department of Genetics and Genome Sciences, Histology Core, Case Western Reserve University, Cleveland, OH USA; 8 Shengli Clinical College, Fujian Medical University, Fuzhou, Fujian China; 9 Department of Health Management, Fujian Provincial Hospital, Fuzhou, Fujian China; 10 Fuzhou University Affiliated Provincial Hospital, Fuzhou, Fujian China

**Keywords:** Pien Tze Huang, Azoxymethane (AOM)/dextran sodium sulfate (DSS), Colitis-associated colorectal cancer, Wnt/β-catenin signaling pathway, RNA-seq

## Abstract

**Background:**

Pien Tze Huang (PZH), a traditional Chinese medicine formulation, is recognized for its therapeutic effect on colitis and colorectal cancer. However, its protective role and underlying mechanism in colitis-associated colorectal cancer (CAC) remain to be elucidated.

**Methods:**

A CAC mouse model was established using AOM/DSS. Twenty mice were randomly divided into four groups (*n* = 5/group): Control, PZH, AOM/DSS, and AOM/DSS + PZH groups. Mice in the PZH and AOM/DSS + PZH group were orally administered PZH (250 mg/kg/d) from the first day of experiment, while the control and AOM/DSS group received an equivalent volume of distilled water. Parameters such as body weight, disease activity index (DAI), colon weight, colon length, colon histomorphology, intestinal tumor formation, serum concentrations of pro-inflammatory cytokines, proliferation and apoptosis in colon tissue were assessed. RNA sequencing was employed to identify the differentially expressed transcripts (DETs) in colonic tissues and related signaling pathways. Wnt/β-Catenin Pathway-Related genes in colon tissue were detected by QPCR and immunohistochemistry (IHC).

**Results:**

PZH significantly attenuated AOM/DSS-induced weight loss, DAI elevation, colonic weight gain, colon shortening, histological damage, and intestinal tumor formation in mice. PZH also notably decreased serum concentration of IL-6, IL-1β, and TNF-α. Furthermore, PZH inhibited cell proliferation and promote apoptosis in tumor tissues. RNA-seq and KEGG analysis revealed key pathways influenced by PZH, including Wnt/β-catenin signaling pathway. IHC staining confirmed that PZH suppressed the expression of β-catenin, cyclin D1 and c-Myc in colonic tissues.

**Conclusions:**

PZH ameliorates AOM/DSS-induced CAC in mice by suppressing the activation of Wnt/β-catenin signaling pathway.

## Introduction

Globally, colorectal cancer (CRC) stands as one of the predominant malignant growths in the digestive system. Data from 2020 indicates that there were approximately 19.3 million new diagnoses and 10 million fatalities due to CRC, placing it third in incidence and second in mortality among all cancers [1]. In China, the latest national cancer statistics released in 2020 indicated that there were 555,000 new cases and 286,000 deaths from CRC, ranking third and fifth among all tumors, respectively [2]. It is evident that the high incidence and mortality rates of CRC are still at elevated levels, posing a significant threat to human health. Therefore, it’s imperative to discover more potent preventive and therapeutic approaches urgently.

The occurrence and development of CRC are associated with various factors, including changes in dietary structure, imbalances in the intestinal microbiota, and inflammatory bowel disease (IBD) [3]. IBD plays a crucial role in the occurrence and progression of CRC. Colitis-associated colorectal cancer (CAC) is a typical type of CRC that evolves from inflammation (specifically, IBD) to tumor transformation. In the context of intestinal inflammation, the intestinal mucosal epithelium undergoes low-grade dysplasia, which then progresses to high-grade dysplasia and eventually breaches the basement membrane to become invasive cancer. This process is referred to as the “inflammation-dysplasia-cancer” pathway of CAC [4, 5]. CAC is characterized by rapid disease progression, a high degree of malignancy, a low 5-year survival rate, and limited treatment options [6]. Research suggests that patients with IBD face a notably higher risk of cancer development compared to those without colitis [7]. This risk is closely related to the degree, duration, and severity of the inflammation [8]. Therefore, there is an urgent need to identify better early prevention and treatment strategies for the progression of colitis-associated colorectal cancer.

In China, natural products, including traditional Chinese medicine, have been historically employed in the clinical treatment of inflammatory diseases due to their minimal side effects [9–11], and are also widely used clinically as adjunctive therapies for CRC [12]. Pien Tze Huang (PZH) is a famous traditional Chinese medicine formula with a history of hundreds of years, which has been employed in the clinical treatment for a variety of diseases in China and Southeast Asia [13, 14]. Recent clinical research has demonstrated that PZH can alleviate pain, improve quality of life, strengthen immunity, and extend the lifespan of patients undergoing treatment for malignant tumors, including liver cancer, CRC [15–18]. The previous in vivo and in vitro studies of our research group have confirmed that PZH can inhibit the growth and metastasis of CRC cells by regulating multiple signaling pathways [19–21]. Notably, our recent research has found that PZH not only alleviates the occurrence and development of DSS-induced ulcerative colitis in mice, but also inhibits the formation and growth of intestinal tumors in AOM/DSS-induced CAC mouse model [22, 23]. These results suggest that PZH has a potential preventive and therapeutic effect on the “inflammation-cancer” transformation of IBD. However, the underlying molecular mechanism of its effects are still to be elucidated. Therefore, in this study, the CAC model mice induced by AOM/DSS and RNA-seq analysis were used to further explores the preventive and therapeutic effects of PZH on CAC, as well as its potential molecular mechanisms of action.

## Materials and methods

### Chemicals and reagents

Azoxymethane (AOM) was purchased from Sigma Chemical Company (St. Louis, MO). Dextran sodium sulfate (DSS, MW: 36 000–50 000) was purchased from MP Biomedicals (Solon, OH, USA). Antigen repair solution, immunohistochemistry kits and phosphate buffered saline were purchased from Maixin Biotechnology (Fuzhou, Fujian, China). Hematoxylin and eosin were purchased from Solarbio Technology Co., Ltd. (Beijing, China). TUNEL kit was purchased from Boster Biological Technology Co., Ltd. (Wuhan, Hubei, China). IL-6, IL-1β and TNF-α ELISA kits were obtained from Proteintech Technology Co., Ltd. (Wuhan, Hubei, China). Primary antibodies specific to PCNA, β-catenin, CyclinD1 and c-Myc were provided by Cell Signaling Technology (Beverly, MA, USA). RNAiso Plus reagent was provided by Takara Biomedical Technology (Beijing) Co., Ltd. (Beijing, China). Ribo-Zero Magnetic Kit (Thermo Fisher Scientific, Grand Island, NY, United States). NEBNext Ploy(A) mRNA Magnetic Isolation Module Kit, NEBNext Ultra RNA Library Prep Kit (New England Biolabs, Beijing, China).

### Preparation of Pien Tze Huang (PZH)

PZH was obtained from and authenticated by the sole manufacturer Zhangzhou Pien Tze Huang (Lot:1806066) Pharmaceutical Company Limited, China. 50 mg of PZH powder was mixed with 1 ml saline and subject to sonication for 30 min. It was ready for intragastric infusion when the powder was completely dissolved, and the solution became clear.

### Animals and experimental protocols

Male C57BL/6 mice (20–22 g) were purchased from Shanghai SLAC Laboratory Animal Co. and housed in a specific pathogen-free facility. All animal care and experimental procedures strictly adhered to the ‘Guide for the Care and Use of Laboratory Animals’ and the ‘Principles for the Utilization and Care of Vertebrate Animals.’ The Committee of Fujian University of Traditional Chinese Medicine (Fujian, China) granted approval for these practices.

**Fig. 1:**
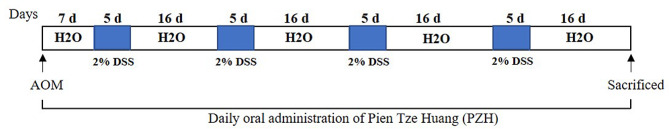
Establishment of a mouse model of AOM/DSS-induced CAC and experimental design

Schematic diagram of the modeling is shown in Fig. 1. Briefly, AOM (12.5 mg/kg) was intraperitoneally injected into the mice at the first day of modeling. After 7 days, mice were provided with 2% DSS in free drinking water for a duration of 5 days. This was replaced with regular drinking water for next 2 weeks. The entire modeling procedure were repeated for four cycles.

Twenty mice were randomly divided into 4 groups (*n* = 5/group). The CAC model in mice was established in both the AOM/DSS group and the AOM/DSS + PZH group, with the exception of the Control and PZH group. From the first day of AOM treatment to the end of the experiment, PZH 250 mg/kg/d or equal volume distilled water were oral gavaged to mice. The dose of PZH used here was from our previously publication. The development of colitis was observed without knowledge of the treatment groups, and included the tracking of body weight, assessment of stool firmness, and detection of rectal bleeding using guaiac paper. The disease activity index (DAI) was determined by a combined score of weight loss, stool consistency, and incidence of rectal bleeding (Table 1).

**Table 1:** Disease activity index (DAI)

Index	Weight loss (%)	Stool	Crypt damage
0	None	Well-formed pellets	None
1	1–5	-	-
2	6–10	Pasty and semiformed	Positive bleeding
3	11–20	-	-
4	> 20	Liquid	Gross bleeding

### Cytokine enzyme-linked immunosorbent assay (ELISA)

At the end of the experiment, blood from each mouse was collected via right heart ventricle puncture. The level of inflammatory cytokines IL-6, IL-1β and TNF-α in the serum was measured using standard ELISA assay diagnostic kits (Proteintech, Chicago, USA) following the manufacturer’s instructions. All the samples were analyzed in triplicate.

### Histopathological analysis

Colon sections of each mouse were stained with hematoxylin and eosin (H&E) as previously described [24]. Briefly, cross-sectional samples of colon tissue from each mouse were preserved in 4% paraformaldehyde (pH 7.4) for a 24-hour period, then processed and set in paraffin before being sliced into sections 4 micrometers thick. After the processes of dewaxing and dehydration, the sections were stained with hematoxylin for 60 s, followed by exposure to 1% hydrochloric acid ethanol for 3 s, and subsequently stained with eosin for 10 s. After mounting the tissues on slides with coverslips, they were examined and imaged at 100× and 200× magnification using a Leica DM400B microscope (Leica, Wetzlar, Germany).

### Immunohistochemistry

Following the removal of wax and rehydration, colon tissue slides of 4 micrometers in thickness were treated with heat to retrieve antigens. Immunohistochemistry (IHC) staining was then carried out on these sections using the Ultra-Sensitive S-P Detection Kit (Maixin), in accordance with the instructions provided by the manufacturer. The paraffin-embedded tissue sections were left to incubate with antibodies at 4° C overnight. This was followed by the addition of HRP-labeled secondary antibody, and then a streptavidin-alkaline phosphatase solution was introduced for reaction. DAB was used for color development, and hematoxylin was applied for counterstaining. After adding a coverslip to each slide, the tissues were examined at a 400× magnification using a Leica DM400B microscope. (Leica, Wetzlar, Germany). Two experienced pathologists, blind to the clinical and pathological details, independently assessed the staining intensity and the proportion of cells that showed positive staining in five distinct fields of each sample. Staining intensity was rated on a scale of 0 to 3: 0 indicating no staining, 1 for faint, 2 for moderate, and 3 for pronounced staining. The percentage of positively stained cells was grouped into four ranges: 1 for 0-25%, 2 for 26-50%, 3 for 51-75%, and 4 for 76-100% [24]. The final protein expression score was calculated by multiplying the values for intensity and percentage.

### TUNEL assay

Using the terminal deoxynucleotidyl transferase dUTP nick end labeling (TUNEL) method, apoptotic cells in the tissue sample were detected in accordance with the manufacturer’s instructions. Briefly, Paraffin-embedded sections were first subjected to standard dewaxing and rehydration procedures. Subsequently, they were treated with 20 µg/ml proteinase K at room temperature for 10 min. A 50 µl labeling reaction mixture, consisting of 5 µl of TdT enzyme and 45 µl of labeling safety buffer (pre-cooled on ice), was then applied to the sections at 37° C in a humidified environment for 60 min and then the reaction was stopped with PBS. The percentage of TUNEL-positive cells and staining intensity were assessed based on the criteria outlined in the “Immunohistochemistry” section.

### RNA sequencing (RNA-Seq) and Kyoto Encyclopedia of Genes and Genomes (KEGG) analyses

Colon tissues were selected from each group (*n* = 5) and their total RNA were extracted using RNAiso Plus reagent. The concentration and quality of RNA was evaluated using the Qubit 3.0 and Agilent 2100 Bioanalyzer. RNA samples with a RIN value of seven or above were used for further experiments.

The construction of the RNA sequencing (RNA-seq) library was facilitated by CapitalBio Technology (Beijing, China). In brief, rRNA was removed from total RNA by Ribo-Zero Magnetic Kit according to the instructions. The resultant rRNA-depleted total RNA was subsequently fragmented, generating the poly(A)-tailed mRNA molecules through NEBNext Ploy(A) mRNA Magnetic Isolation Module Kit according to the manufacturer’s instructions. The development of sequencing libraries was executed using the NEBNext Ultra RNA Library Prep Kit for Illumina, strictly following the manufacturer’s guidelines.

After sequencing, the raw data were processed by a bioinformatics workflow, including the following steps. (1) The sequencing quality was assessed by FastQC (v0.11.5), and low-quality data were filtered by NGSQC (v.2.3.3). (2) Clean reads were aligned to the mouse genome (GCRmm38/mm10 in UCSC) by HISAT2 (v2.1.0) with standard paraments. (3) The reconstruction and quantification of genes and transcripts were executed utilizing StringTie (v1.3.3b), based on read comparison results. (4) Bio MAS (molecule annotation system v3.0) was used for the correlative analysis between samples and the functional annotation of genes.

The DESeq (v1.28.0) was used for differential expression transcripts (DETs) analysis, setting a cut-off at > 2 with a significance level of P-value < 0.05. The DETs were identified and analyzed through volcano and hierarchical clustering plots. Subsequent to identifying the most represented signaling pathways among the DETs, a thorough KEGG pathway enrichment analysis was conducted [25]. We uploaded the raw data to the Gene Expression Omnibus (GEO) database (Submission No.: GSE230302).

### Quantitative real-time Reverse Transcription Polymerase Chain Reaction (qRT-PCR)

Total RNA was extracted from colon tissue using RNAiso Plus reagent (Takara, Beijing, China). The total RNA was quantified using NanoDrop 2000 (Thermo Fisher, USA). Reverse transcription into complementary DNA (cDNA) was performed according to the manufacturer’s instructions using the PrimeScript RT reagent kit (Takara). The cDNA was then amplified on a quantitative PCR instrument according to the TB Green^®^ Premix Ex Taq (Takara). The process of the PCR reaction was initiated at 95 ◦C for 30 s, followed by 40 cycles (95 ◦C for 5 s and 60 ◦C for 30 s). Relative mRNA levels were calculated using the comparative method (2^ΔΔCt^), with GAPDH serving as an internal reference gene. The primer sequences (General Biol, China) used were listed in Table 2.

**Table 2:** Primer sequences used in qRT-PCR

Genes	Primer	Sequence (5’-3’)
Jun	Forward	TTCCTCCAGTCCGAGAGCG
	Reverse	TGAGAAGGTCCGAGTTCTTGG
Wif1	Forward	CTGGAGCATCCTACCTTGCC
	Reverse	GATGGGCGTCGATCCACAG
Axin2	Forward	ATGAGTAGCGCCGTGTTAGTG
	Reverse	GGGCATAGGTTTGGTGGACT
Ctnnb1	Forward	ATGGAGCCGGACAGAAAAGC
	Reverse	TGGGAGGTGTCAACATCTTCTT
Dkk2	Forward	TCAGTCAGCCAACCGATCTG
	Reverse	TCTCTGTGGCATCGTTTCTTTT
Notum	Forward	GGACAGCTTTATGGCGCAAG
	Reverse	TCACCGACGTGTTCAGCAG
Nkd1	Forward	CAGCTTGCTGCATACCATCTAT
	Reverse	GTTGAAAAGGACGCTCCTCTTA
Fzd10	Forward	CATGCCCAACCTGATGGGTC
	Reverse	GCCACCTGAATTTGAACTGCTC
Wnt16	Forward	CAGGGCAACTGGATGTGGTT
	Reverse	CTCGTGTCGGAACTGGCTTC
GAPDH	Forward	GAAGGGTGGAGCCAAAAGG
	Reverse	CTTCTGGGTGGCAGTGATGG

### Statistics analysis

Data analysis was performed with the SPSS 26.0 software. For assessing differences among multiple groups, we either applied one-way ANOVA or the Kruskal-Wallis H test. Quantitative data was presented as the mean, accompanied by the standard deviation (SD). A two-sided P-value of less than 0.05 was considered statistically significant. All experiments were replicated a minimum of three times to ensure reliability.

## Results

### Effects of PZH on body weight and DAI in CAC mouse model

**Fig. 2:**
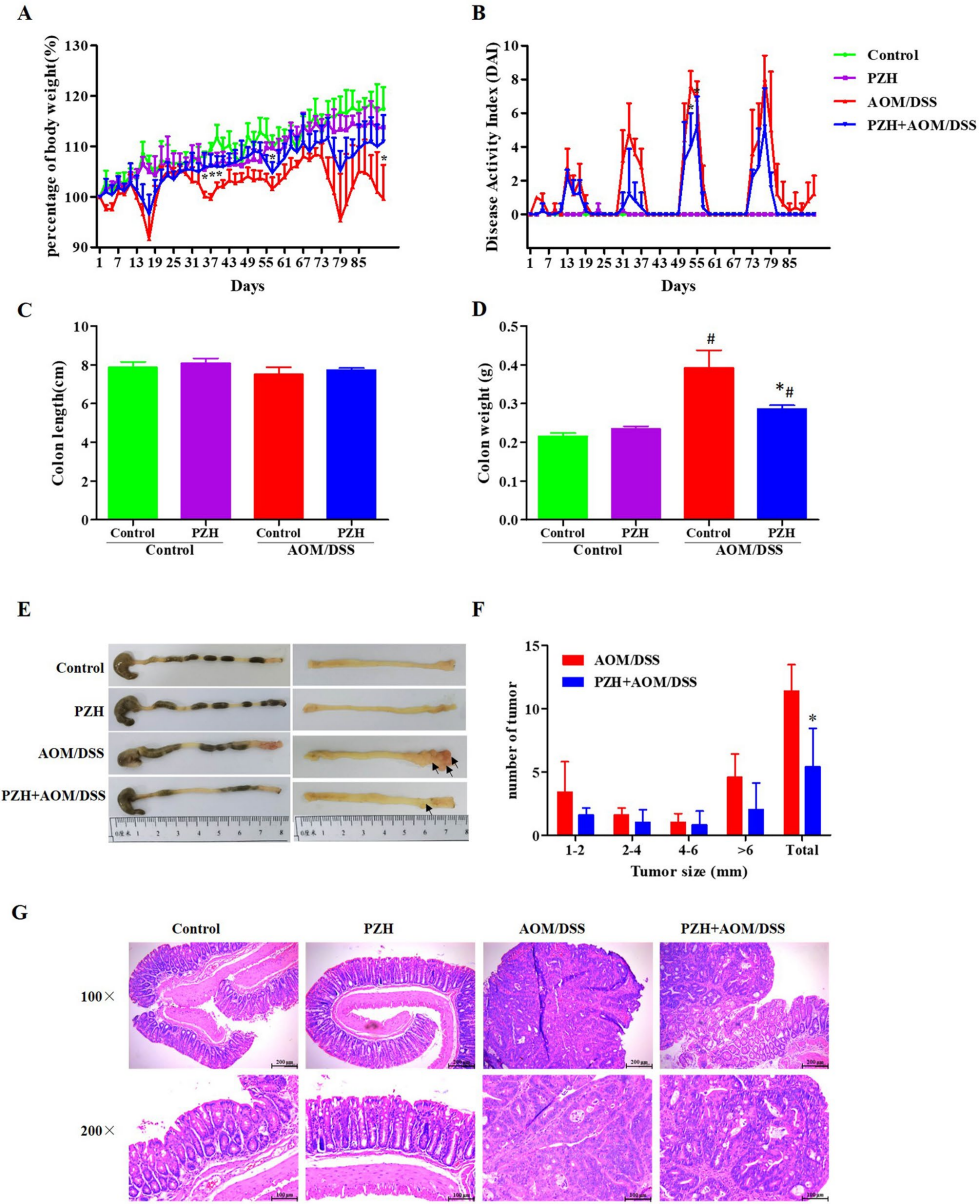
Effect of Pien Tze Huang (PZH) on AOM/DSS-induced CAC in mice. (**A**) Body weight changed curve of mice in each group. (**B**) DAI score changed curve of mice in each group. (**C**) Weight of colon. (**D**) Length of colon. (**E**) Representative histopathological images. (**F**) Number of colon tumors. (**G**) Representative photographs of HE staining. *P < 0.05, vs. the Control group, #*P* < 0.05, vs. the AOM/DSS group

This study first investigated the effects of PZH on body weight and DAI of AOM/DSS-induced CAC mouse models. The results presented in Fig. 2A, the weight of the mice in Control group and PZH group showed a slow upward trend, and there was no notable difference between two groups. However, the body weight of mice in AOM/DSS group and PZH + AOM/DSS group fluctuated periodically with the cycle of 2% DSS administration, which was consistent with the signs of 2% DSS administration. Compared with the AOM/DSS group, the weight loss of PZH + AOM/DSS group was alleviated, and the difference was statistically significant during the second and third cycles of administration. (*P < 0.05, Fig. 2A). As depicted in Fig. 2B, the DAI of mice was 0 of in the Control group and the PZH group. However, the DAI of AOM/DSS + PZH group exhibited a slightly lower than AOM/DSS group during the 2% DSS administration, especially the difference was statistically significant during the third cycle of administration (*P < 0.05).

### Effects of PZH on colon weight and length in CAC mouse model

The colon length and weight of mice were measured at the end of the experiment shown in Fig. 2C-D. No significant differences were observed in the colon lengths among the four groups (Fig. 2C). The weights of the colons in the Control and PZH groups were 0.215 ± 0.020 g and 0.234 ± 0.02 g, respectively, and the difference was not statistically significant. However, the weight of the colon in the PZH + AOM/DSS group (0.286 ± 0.02 g) was significantly lower than that in the AOM/DSS group (0.392 ± 0.10 g) (*P < 0.05, Fig. 2D).

### Effects of PZH on the number of intestinal tumors in CAC model mice

Further observations on the formation of intestinal tumors in CAC model mice revealed that both the Control and PZH groups exhibited clear fecal pellets in the large intestine, with a smooth intestinal lining devoid of any noticeable thickening or tumor formation (Fig. 2E-F). In contrast, mice in AOM/DSS and PZH + AOM/DSS groups displayed evident tumor formation within the intestine, predominantly localized at the distal end of the colon, with the highest incidence near the anal segment (indicated by black arrows) (Fig. 2E). The average number of tumors formed in the AOM/DSS group was 11.4 per mouse. However, the PZH + AOM/DSS group exhibited a significantly lower average number of tumors (5.4 per mouse) compared to the AOM/DSS group (*P < 0.05, Fig. 2F). These results suggest that PZH treatment can inhibit the formation and growth of tumors in the colon of CAC model mice induced by AOM/DSS.

### Effects of PZH on pathological damage of colon tissue in CAC model mice

Figure 2G showed the pathological changes in the colon tissues of mice from each group, as detected by HE staining. The colon tissues of the Control and PZH groups both exhibited intact structures, with epithelial cells neatly arranged in a columnar shape, nuclei located in the middle to lower part of the cells, and multiple scattered goblet cells visible. No cancer cells or inflammatory cells were observed. In the AOM/DSS and PZH + AOM/DSS groups, different degrees of inflammatory cell infiltration were observed in the non-tumor parts of the colon tissues, with disordered and deformed epithelial cells, disappearance of crypts, reduction of goblet cells, and visible adenoma formation. However, compared to the AOM/DSS group, the PZH + AOM/DSS group showed a reduction in the tumor area and a significant alleviation of pathological damage in the colon tissue. These results suggest that PZH intervention can alleviate the pathological damage of colon tissue in CAC model mice.

### Effects of PZH on serum inflammatory cytokine levels in CAC model mice

**Fig. 3:**
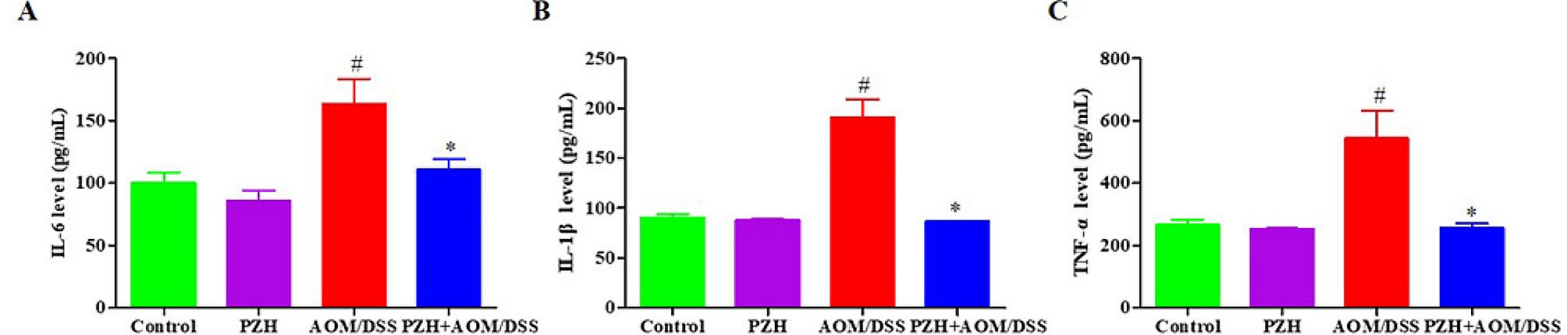
Effect of Pien Tze Huang (PZH) on the levels of inflammatory mediators on AOM/DSS-induced colon cancer in mice. (**A**) IL-6; (**B**) IL-1β; (**C**) TNF-α. *P < 0.05, vs. the Control group, #*P* < 0.05, vs. the AOM/DSS group

To investigate the effects of PZH on pro-inflammatory cytokines in CAC model mice, this study utilized ELISA to determine the concentrations of IL-6, TNF-α, and IL-1β in the serum of mice. The results are presented in Fig. 3. No notable differences were observed in these inflammatory cytokines’ concentration between the Control and PZH groups. Serum concentration of IL-6 (Fig. 3A), TNF-α (Fig. 3B), and IL-1β (Fig. 3C) in the AOM/DSS group were notably elevated compared to the Control and PZH groups (#*P* < 0.05). On the other hand, the concentrations of IL-6, TNF-α and IL-1β in the serum of the PZH + AOM/DSS group were significantly reduced compared to the AOM/DSS group (*P < 0.05). These results suggest that PZH can inhibit the secretion of inflammatory cytokines in the serum of CAC model mice, thereby suppressing the inflammatory response.

### Effects of PZH on cell proliferation and apoptosis in colon tissue of CAC model mice

**Fig. 4:**
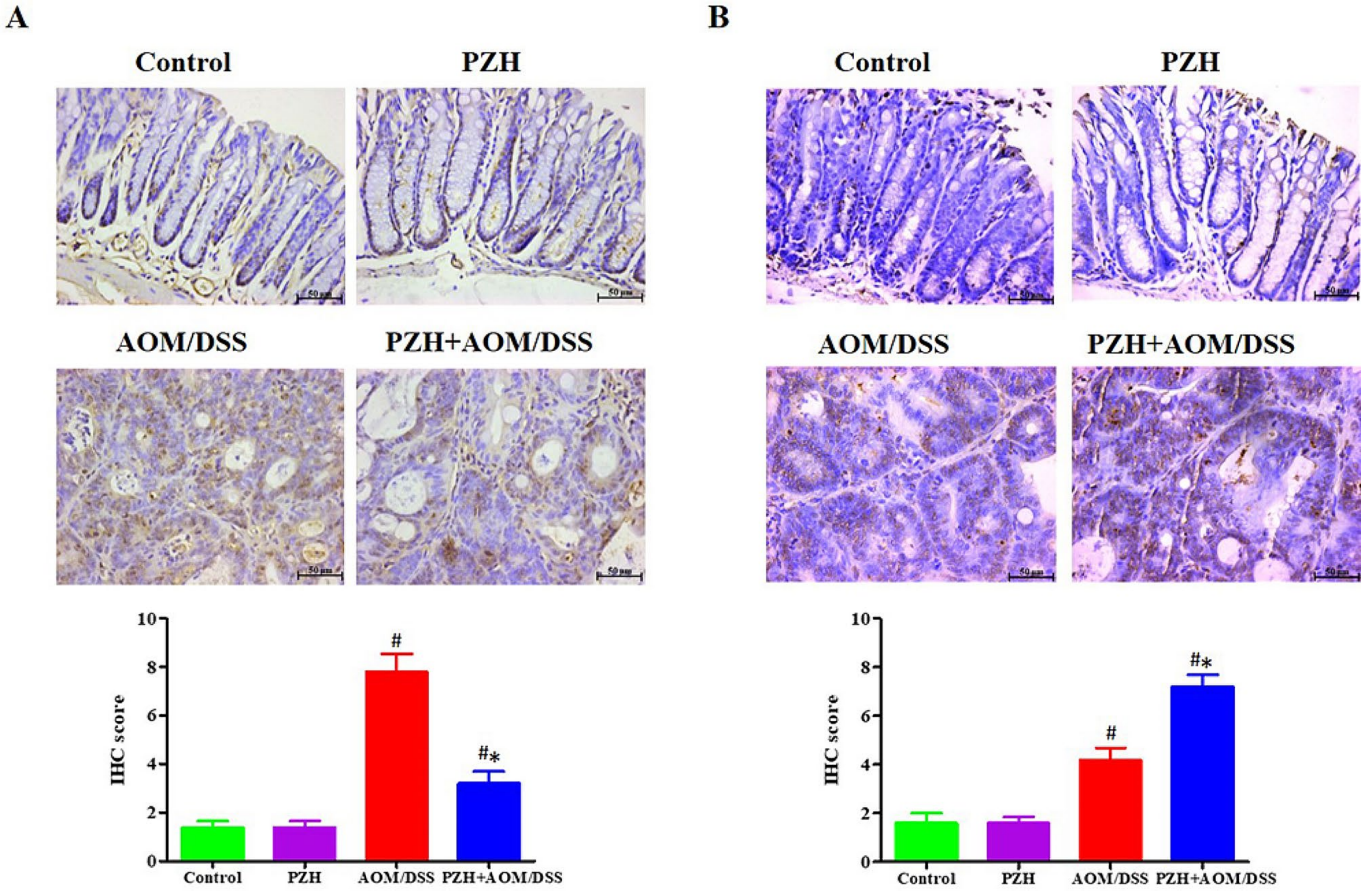
Effect of Pien Tze Huang (PZH) on the expression of PNCA and apoptosis on CAC mice. IHC was performed to detect (**A**) PCNA expression in colon tissues, and (**B**) TUNEL staining was used to determine the apoptotic cells in tissues of each group. The representative images of IHC analysis or TUNEL staining were taken at a magnification of 400× (left panel) and IHC scores were calculated (right panel). *P < 0.05, vs. the Control group, #*P* < 0.05, vs. the AOM/DSS group

To explore the effects of PZH on cell proliferation and apoptosis in the colon tissue of CAC model mice, immunohistochemistry was firstly employed to detect the expression of PCNA protein in the colon tissue. The results are shown in Fig. 4A. Both the Control and PZH groups exhibited low levels of PCNA expression in the colon tissue, with no significant differences. The expression of PCNA in the tumor tissue of the colon in the AOM/DSS group was significantly higher than that in the Control and PZH groups (#*P* < 0.05). Conversely, the expression of PCNA in the colon tissue of the PZH + AOM/DSS group was significantly lower than that in the AOM/DSS group (*P < 0.05). Further analysis using the TUNEL assay revealed the number of apoptotic cells in the colon tissue of each group, as shown in Fig. 4B. Both the Control and PZH groups had fewer apoptotic cells in the colon tissue, with no significant differences. The number of apoptotic cells in the colon tissue of the AOM/DSS and PZH + AOM/DSS groups was significantly increased (#*P* < 0.05), but the PZH + AOM/DSS group had significantly fewer apoptotic cells than the AOM/DSS group (*P < 0.05). These results indicate that PZH intervention can inhibit excessive cell proliferation and promote apoptosis in tumor tissues, thereby suppressing tumor cell growth.

### Effects of PZH on gene expression in colon tissue of CAC model mice

**Fig. 5:**
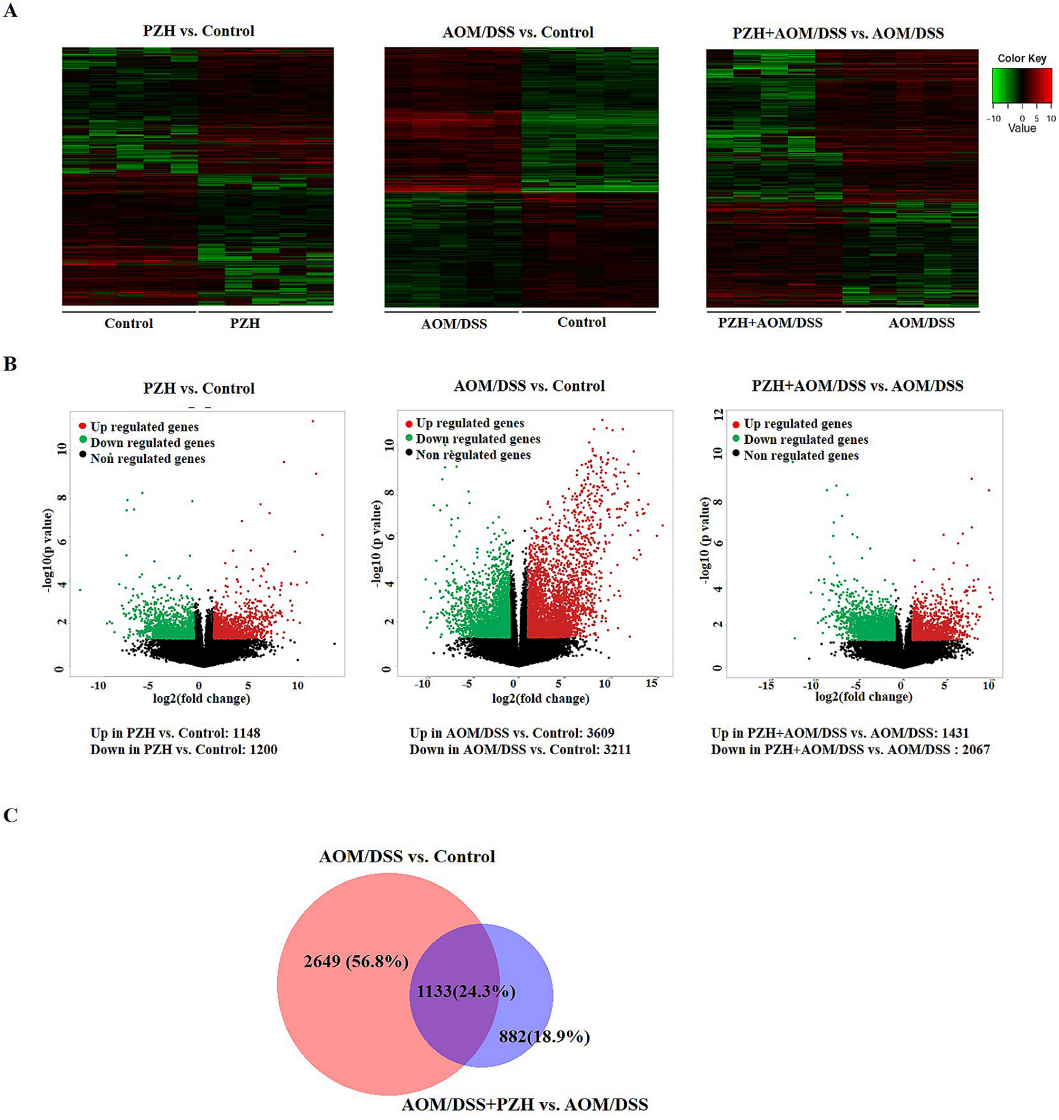
Genome-wide gene expression profiling in the colon tissue of mice. (**A**) Hierarchical clustering plots and (**B**) Volcano plots were used to compare gene expression profiles (|fold change| ≥2, *P* < 0.05). (**C**) The overlapping area represents genes in the AOM/DSS group and in the AOM/DSS + PZH group (*n* = 1133)

To explore the potential molecular mechanisms underlying the protective effects of PZH against CAC, this study further employed high-throughput RNA sequencing technology to analyze the differential gene expression in the colon tissues of each group of mice. A Fold Change > 2 and P-value < 0.05 were used as the screening criteria. The results comparing the number of differentially expressed genes between each group are shown in Fig. 5. Compared to the Control group, the PZH group had 2348 differentially expressed transcripts, of which 1148 were upregulated and 1200 were downregulated. Compared to the Control group, the AOM/DSS group had 6420 differentially expressed transcripts, of which 3609 were upregulated and 3211 were downregulated. Compared to the AOM/DSS group, the PZH + AOM/DSS group had 3498 differentially expressed transcripts, of which 1431 were upregulated and 2067 were downregulated. Based on the hypothesis that the differentially expressed genes in the colon tissue of CAC model mice induced by AOM/DSS and intervened by PZH could be key genes in the inhibition of CAC development by PZH, the differentially expressed transcripts between the AOM/DSS and Control groups and between the PZH + AOM/DSS and AOM/DSS groups were intersected, resulting in 1133 (24.3%) differentially expressed transcripts. This suggests that these differentially expressed genes may be potential functional genes involved in the inhibitory effects of PZH on the development of CAC.

### Effects of PZH on differentially expressed gene-related pathways in colon tissue of CAC model mice

**Fig. 6:**
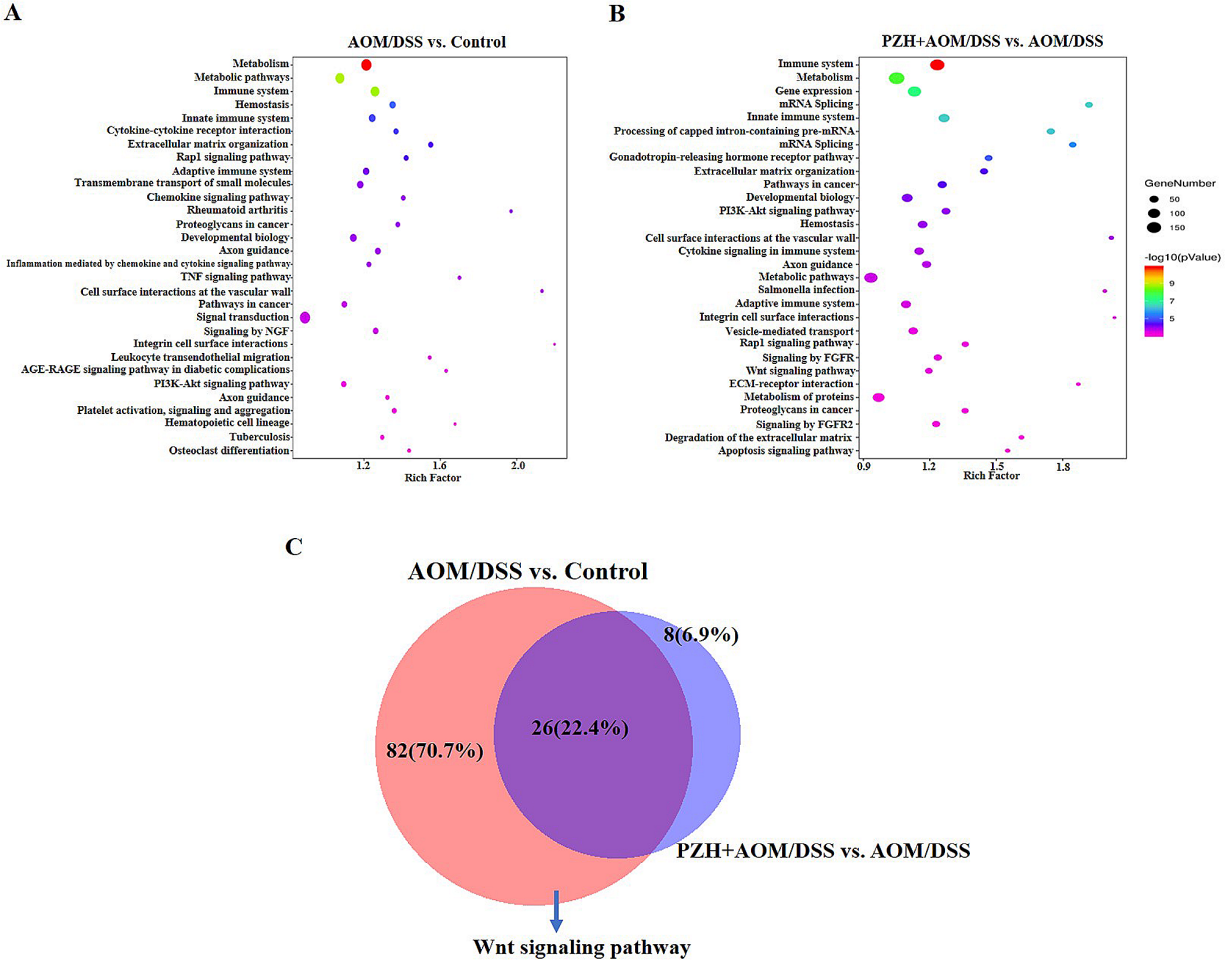
KEGG signaling pathways in the colon tissue of mice. (**A**-**B**) KEGG pathway enrichment analysis of differentially expressed transcripts in the two comparisons of AOM/DSS group vs. control group and AOM/DSS + PZH group vs. AOM/DSS group. (**C**) The overlapping KEGG pathways in the two comparisons of AOM/DSS group vs. Control group and of AOM/DSS + PZH group vs. AOM/DSS group

Further enrichment analysis was conducted on the differentially expressed genes in each group to identify potential pathways that may be regulated by PZH in inhibiting the development of CAC. A P-value < 0.001 was used as the screening criterion. The enrichment results are shown in Fig. 6. A total of 108 pathways were effectively enriched in the differentially expressed genes between the AOM/DSS and Control groups, mainly focusing on the immune system, metabolism, PI3K-AKT signaling pathway, and extracellular matrix, among other signaling pathways (Fig. 6A). A total of 34 pathways were effectively enriched in the differentially expressed genes between the PZH + AOM/DSS and AOM/DSS groups, mainly focusing on the extracellular matrix, Wnt signaling pathway, and cancer pathways, among others (Fig. 6B; Table 3). The intersection of the enriched pathways from both comparisons yielded 26 common pathways, accounting for 22.4%, including the Wnt signaling pathway, PI3K-AKT signaling pathway, pathways in cancer, and metabolic pathways (Fig. 6C).

**Table 3:** Overlapping enriched pathways between AOM/DSS group vs. Control group and AOM/DSS + PZH group vs. AOM/DSS group

No.	#Term	Database	ID
1	Immune System	Reactome	R-MMU-168,256
2	Metabolism	Reactome	R-MMU-1,430,728
3	Innate Immune System	Reactome	R-MMU-168,249
4	Extracellular matrix organization	Reactome	R-MMU-1,474,244
5	Pathways in cancer	KEGG PATHWAY	mmu05200
6	Developmental Biology	Reactome	R-MMU-1,266,738
7	PI3K-Akt signaling pathway	KEGG PATHWAY	mmu04151
8	Hemostasis	Reactome	R-MMU-109,582
9	Cell surface interactions at the vascular wall	Reactome	R-MMU-202,733
10	Cytokine Signaling in Immune system	Reactome	R-MMU-1,280,215
11	Axon guidance	Reactome	R-MMU-422,475
12	Metabolic pathways	KEGG PATHWAY	mmu01100
13	Salmonella infection	KEGG PATHWAY	mmu05132
14	Adaptive Immune System	Reactome	R-MMU-1,280,218
15	Integrin cell surface interactions	Reactome	R-MMU-216,083
16	Rap1 signaling pathway	KEGG PATHWAY	mmu04015
17	Signaling by FGFR	Reactome	R-MMU-190,236
18	Wnt signaling pathway	PANTHER	P00057
19	ECM-receptor interaction	KEGG PATHWAY	mmu04512
20	Proteoglycans in cancer	KEGG PATHWAY	mmu05205
21	Signaling by FGFR2	Reactome	R-MMU-5,654,738
22	Degradation of the extracellular matrix	Reactome	R-MMU-1,474,228
23	Metabolism of lipids and lipoproteins	Reactome	R-MMU-556,833
24	Signaling by PDGF	Reactome	R-MMU-186,797
25	Fc gamma R-mediated phagocytosis	KEGG PATHWAY	mmu04666
26	phospholipases	BioCyc	LIPASYN-PWY

### Effects of PZH on differentially expressed genes of Wnt signaling pathway in colon tissue of CAC model mice

**Fig. 7:**
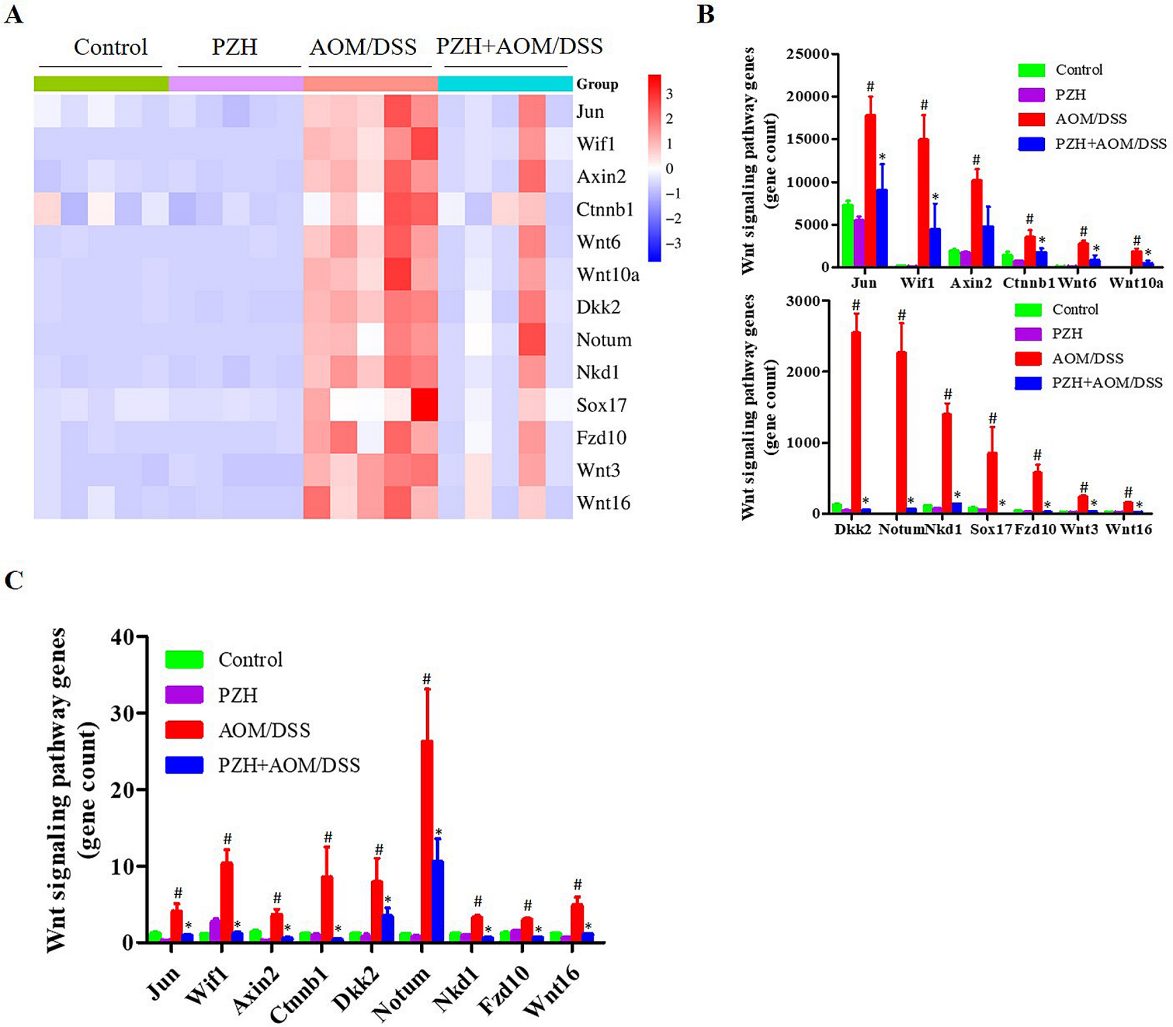
The differentially expressed genes in the Wnt pathways. (**A**) The hierarchical cluster analysis from RNA sequencing data. (**B**) The column chart analysis from RNA sequencing data. (**C**) The relative mRNA expressions as determined by qRT-PCR. *P < 0.05, vs. the Control group, #*P* < 0.05, vs. the AOM/DSS group

Compared with the Control and PZH groups, the expressions of Jun, Wif1, Axin2, Ctnnb1, Wnt6, Wnt10a, Dkk2, Notum, Nkd1, Sox17, Fzd10, Wnt3 and Wnt16 were significantly up-regulated in AOM/DSS group (#*P* < 0.05). Compared with the AOM/DSS group, the expressions of Jun, Wif1, Axin2, Ctnnb1, Wnt6, Wnt10a, Dkk2, Notum, Nkd1, Sox17, Fzd10, Wnt3, Wnt16 were observably down-regulated in PZH treatment group(*P < 0.05) (Fig. 7A-B). To verify the reliability of RNA sequencing results, 9 genes from Wnt signaling pathways were selected and validated by qRT-PCR. The qRT-PCR results showed that the mRNA expressions of Jun, Wif1, Axin2, Ctnnb1, Dkk2, Notum, Nkd1, Fzd10 and Wnt16 were significantly up-regulated in the AOM/DSS group compared with the Control and PZH groups, while PZH treatment significantly down-regulated these mRNA expressions (Fig. 7C). These results were consistent with the RNA sequencing results.

### Effects of PZH on the expression of Wnt/β-Catenin pathway-related proteins in colon tissue of CAC Model mice

**Fig. 8:**
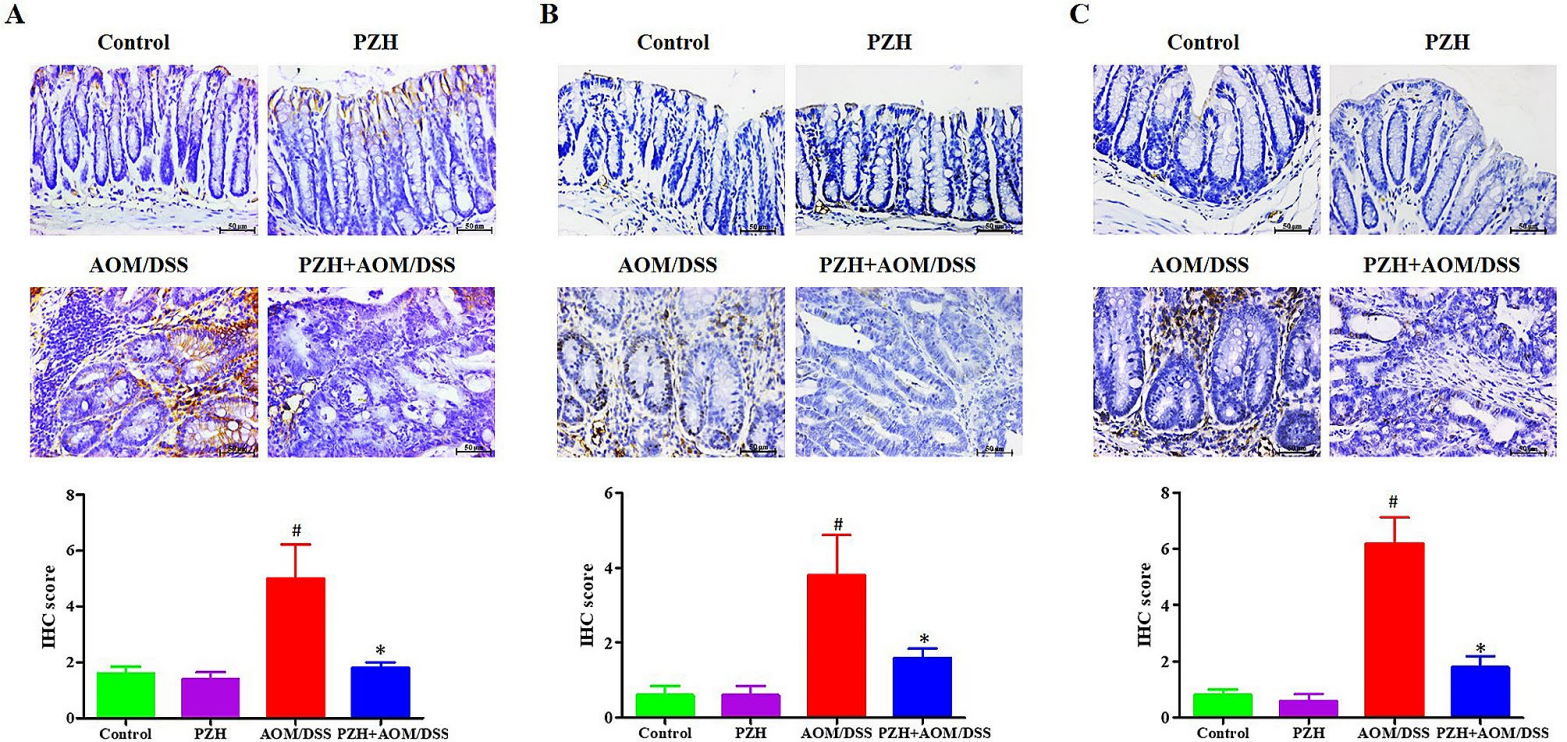
Effect of Pien Tze Huang (PZH) on the Wnt/β-catenin signaling pathway on AOM/DSS-induced colon cancer in mice. IHC was performed to detect (**A**) β-catenin and (**B**) cyclinD1 and (**C**) c-Myc expression in colon tissues of each group. The representative images of IHC analysis were taken at a magnification of 400× (top panel) and IHC scores were calculated (below panel). *P < 0.05, vs. the Control group, # *P* < 0.05, vs. the AOM/DSS group

A view of the key role of the Wnt/β-catenin signaling pathway in colon cancer progression and its potential for long-term activation to drive CAC development [26], this study further employed the IHC method to verify the expression of β-catenin and its downstream proteins Cyclin D1 and c-Myc in mouse colon tissue. The results are shown in Fig. 8. The expression of β-catenin in both the Control and PZH groups was relatively low, with no significant differences. The expression of β-catenin in the colon tissue of mice in the AOM/DSS group was significantly higher than that in the Control and PZH groups (#*P* < 0.05); whereas the expression of β-catenin in the PZH + AOM/DSS group was significantly lower than that in the AOM/DSS group (*P < 0.05) (Fig. 8A). As expected, the expression of downstream proteins Cyclin D1 and c-Myc showed the same trend as the expression of β-catenin (Fig. 8B-C). These results further confirm that PZH may inhibit the development of CAC by suppressing the activity of the Wnt/β-catenin signaling pathway.

## Discussion

CRC is a major public health challenge in the real of malignant diseases [1]. Ulcerative colitis is among the highest-risk populations for developing colorectal cancer [13]. CAC originates from inflamed mucosa and is the result of activated inflammatory responses and cancer progression cascades [27]. Clinical studies have shown that, compared to the general population, individuals with IBD have a 19-fold increased risk of developing CAC [13]. Therefore, early intervention and prevention of the “inflammation-to-cancer” transition in IBD serve as effective strategies for the prevention and treatment of CAC.

In China, traditional Chinese medicine is widely adopted as an adjunctive therapy by a large number of CRC patients [12]. Existing clinical studies have also confirmed that PZH has effects such as relieving pain and improving patient prognosis in the prevention and treatment of ovarian cancer and colorectal cancer [15–19]. Our previous research has found that PZH can inhibit the formation of intestinal tumors in a mouse model of CAC [20]. Therefore, this study used a CAC mouse model induced by the combined use of AOM and DSS to initially explore the effect of PZH on the formation and growth of intestinal tumors in the CAC mouse model. The results found that PZH not only inhibited the increase in the number and volume of intestinal tumors in CAC mice but also suppressed the secretion of pro-inflammatory cytokines IL-6, TNF-α, and IL-1β in mouse serum. Moreover, PZH alleviated the pathological damage of mouse colon tissue and inhibited tumor cell proliferation while promoting cell apoptosis. These results demonstrated that PZH may inhibit the growth of tumor cell in CAC mice by suppressing abnormal cell proliferation and promoting cell apoptosis.

Previous research has found that the development of CAC is not only closely related to the abnormal expression and mutation of various genes such as TP53, IL-10, HMGB1 [28–30], but also closely related to the abnormal activation of multiple signaling pathways such as Wnt/β-catenin, NF-kB [31, 32]. These could potentially become key targets and pathways to prevent the “inflammation-to-cancer” transition in IBD, thereby playing a role in the prevention and treatment of CAC progression. Given that PZH has the characteristics of exerting its anti-tumor effects through multiple components, multiple targets, and multiple pathways, this study further used high-throughput RNA sequencing methods to analyze the impact of PZH on CAC-related genes and pathways, to clarify its potential molecular mechanism of therapeutic action. Using Fold Change > 2 and P-value < 0.05 as screening criteria, differentially expressed genes between each group were compared. The results showed that thousands of transcripts changed in each pairwise comparison. The intersection of differentially expressed transcripts between the AOM/DSS and Control groups and between the PZH + AOM/DSS and AOM/DSS groups resulted in 1133 (24.3%) differentially expressed transcripts, which are likely one of the potential mechanisms by which PZH inhibits the development of CAC. Further enrichment analysis of differentially expressed genes in each group revealed related pathways, using a P-value of 0.001 as the screening criterion. The results showed that differentially expressed gene-related pathways between the AOM/DSS and Control groups were mainly enriched in the immune system, metabolism, PI3K/AKT signaling pathway, extracellular matrix, and other signaling pathways. Differentially expressed gene-related pathways between the PZH + AOM/DSS and AOM/DSS groups were mainly enriched in the extracellular matrix, Wnt signaling pathway, cancer pathways, and other signaling pathways. The intersection of the enriched pathways from both comparisons yielded 26 common pathways, including the Wnt signaling pathway, PI3K/AKT signaling pathway, pathways in cancer, and metabolic pathways.

Research has shown that the aberrant activation of the Wnt/β-catenin signaling pathway is pivotal in the development and progression of colorectal cancer [33]. Its abnormal activation led to nuclear translocation of β-catenin, thereby regulating the transcription of some proliferation-related genes such as Cyclin D1 and c-Myc [34]. Previous investigation has shown the continuous activation of the Wnt/β-catenin signaling pathway as a potential factor in the development of CAC, its exact role in the development and progression of CAC needs further elucidation [35]. Based on the RNA sequencing analysis results of this study, the differentially expressed gene-related pathways mainly enriched in the PZH + AOM/DSS group compared to the AOM/DSS group also included the Wnt/β-catenin signaling pathway. Therefore, this study further verified the RNA sequencing analysis results through immunohistochemistry. As expected, compared to the control group, the AOM/DSS group of mice exhibited a significantly increased in the expression of β-catenin, Cyclin D1, and c-Myc in the colon tissue; whereas the intervention with PZH significantly inhibited the increase in the expression of these proteins. From this, we speculate that PZH may inhibit the development and progression of CAC by regulating the Wnt/β-catenin signaling pathway and thereby suppressing tumor cell proliferation, which could be one of its important mechanisms.

## Conclusion

PZH can significantly alleviate clinical symptoms of AOM/DSS-induced CAC mice, inhibit the formation and growth of tumors in colon by inhibiting the activation of the Wnt/β-catenin signaling pathway. Our findings provide further experimental evidence for the clinical treatment of colorectal cancer with PZH.

## Data Availability

The data sets generated in the current study have been deposited into the NCBI GEO database (GEO No. GSE230302) and data supporting the results are included in this published article.

## Abbreviations

AOM: Azoxymethane
CAC: Colitis-associated colorectal cancer
CRC: Colorectal cancer
DETs: Differentially expressed transcripts
DSS: Dextran sodium sulfate
ELISA: Enzyme-linked immunosorbent assay
GEO: Gene expression omnibus
H&E: Hematoxylin and eosin
IBD: inflammatory bowel disease
IHC: immunohistochemistry
PZH: Pien Tze Huang
TUNEL: Terminal deoxynucleotidyl transferase dUTP nick end labeling

## References

[1] Marshall JR (2008). Prevention of colorectal cancer: diet, chemoprevention, and lifestyle. Gastroenterol Clin North Am.

[2] Xia C, Dong X, Li H, Cao M, Sun D, He S, Yang F, Yan X, Zhang S, Li N (2022). Chen.Cancer statistics in China and United States, 2022: profiles, trends, and determinants. Chin Med J (Engl).

[3] Feagins LA, Souza RF (2009). Spechler.Carcinogenesis in IBD: potential targets for the prevention of colorectal cancer. Nat Rev Gastroenterol Hepatol.

[4] Canavan C, Abrams KR. and J. Mayberry.Meta-analysis: colorectal and small bowel cancer risk in patients with Crohn’s disease.Aliment Pharmacol Ther.2006;23. 1097 – 104. 10.1111/j.1365-2036.2006.02854.x 16611269

[5] Zhang X, Li W, Ma Y, Zhao X, He L, Sun P (2021). Wang.High-fat diet aggravates colitis-associated carcinogenesis by evading ferroptosis in the ER stress-mediated pathway. Free Radic Biol Med.

[6] Wu Y, Yao J, Xie J, Liu Z, Zhou Y, Pan H. and W. Han.The role of autophagy in colitis-associated colorectal cancer.Signal transduct Target Ther.2018;3. 31. 10.1038/s41392-018-0031-8 PMC6265276 30510778

[7] Sanmarco LM, Chao CC, Wang YC, Kenison JE, Li Z, Rone JM, Rejano-Gordillo CM, Polonio CM, Gutierrez-Vazquez C, Piester G, Plasencia A, Li L, Giovannoni F, Lee HG, Faust Akl C, Wheeler MA, Mascanfroni I, Jaronen M, Alsuwailm M, Hewson P, Yeste A, Andersen BM, Franks DG, Huang CJ, Ekwudo M, Tjon EC, Rothhammer V, Takenaka M, de Lima KA, Linnerbauer M, Guo L, Covacu R, Queva H, Fonseca-Castro PH, Bladi MA, Cox LM, Hodgetts KJ, Hahn ME, Mildner A, Korzenik J, Hauser R, Snapper SB. and F.J. Quintana.Identification of environmental factors that promote intestinal inflammation.Nature.2022;611. 801–9. 10.1038/s41586-022-05308-6 PMC9898826 36266581

[8] Niu M, Chong Y, Han Y (2015). Liu.Novel reversible selective inhibitor of nuclear export shows that CRM1 is a target in colorectal cancer cells. Cancer Biol Ther.

[9] Treasure. J.Herbal medicine and cancer: an introductory overview.Semin Oncol Nurs.2005;21. 177 – 83. 10.1016/j.soncn.2005.04.006 16092805

[10] Bensoussan M, Jovenin N, Garcia B, Vandromme L, Jolly D, Bouché O, Thiéfin G (2006). Cadiot.Complementary and alternative medicine use by patients with inflammatory bowel disease: results from a postal survey. Gastroenterol Clin Biol.

[11] Langhorst J, Wulfert H, Lauche R, Klose P, Cramer H, Dobos GJ (2015). Korzenik.Systematic review of complementary and alternative medicine treatments in inflammatory bowel diseases. J Crohns Colitis.

[12] Fang L, Chen B, Liu S, Wang R, Hu S, Xia G, Tian Y (2012). Cai.Synergistic effect of a combination of nanoparticulate Fe3O4 and gambogic acid on phosphatidylinositol 3-kinase/Akt/Bad pathway of LOVO cells. Int J Nanomed.

[13] Li JM, Lee YC, Li CC, Lo HY, Chen FY, Chen YS, Hsiang CY (2018). Pathways J Agric Food Chem.

[14] Cao W, Liu J, Dai Y, Zhou Y, Li R. and P. Yu.Bibliometric Analysis of Marine Traditional Chinese Medicine in Pharmacopoeia of the People’s Republic of China: Development, Differences, and Trends Directions.Evid Based Complement Alternat Med.2022;2022. 3971967. 10.1155/2022/3971967 PMC9810416 36605100

[15] Lü L, Wai MS, Yew DT. and Y.T. Mak.Pien Tze Huang, a composite Chinese traditional herbal extract, affects survival of neuroblastoma cells. Int J Neurosci.2009;119. 255 – 62. 10.1080/00207450802324770 19125378

[16] He F, Wu HN, Cai MY, Li CP, Zhang X, Wan Q, Tang SB (2014). Cheng.Inhibition of ovarian cancer cell proliferation by Pien Tze Huang via the AKT-mTOR pathway. Oncol Lett.

[17] Fu Y, Zhang L, Hong Z, Zheng H, Li N, Gao H, Chen B. and Y. Zhao.Methanolic extract of Pien Tze Huang induces apoptosis signaling in human osteosarcoma MG63 cells via Multiple Pathways.Molecules.2016;21. 283. 10.3390/molecules21030283 PMC6274404 26938521

[18] Chen ZP (2021). Tze Huang (PZH) as a Multifunction Medicinal Agent in Traditional Chinese Medicine (TCM): a review on cellular, molecular and physiological mechanisms. Cancer Cell Int.

[19] Shen A, Chen H, Chen Y, Lin J, Lin W, Liu L, Sferra TJ. and J. Peng.Pien Tze Huang Overcomes Multidrug Resistance and Epithelial-Mesenchymal Transition in Human Colorectal Carcinoma Cells via Suppression of TGF-β Pathway.Evid Based Complement Alternat Med.2014;2014. 679436. 10.1155/2014/679436 PMC4253702 25505925

[20] Shen A, Chen Y, Hong F, Lin J, Wei L, Hong Z, Sferra TJ. and J. Peng.Pien Tze Huang suppresses IL-6-inducible STAT3 activation in human colon carcinoma cells through induction of SOCS3.Oncol Rep.2012;28. 2125–30. 10.3892/or.2012.2067 23027374

[21] Wan Y, Shen A, Qi F, Chu J, Cai Q, Sferra TJ, Peng J (2017). Chen.Pien Tze Huang inhibits the proliferation of colorectal cancer cells by increasing the expression of miR-34c-5p.Exp. Ther Med.

[22] Li L, Shen A, Chu J, Sferra TJ, Sankararaman S, Ke X, Chen Y (2018). Peng.Pien Tze Huang ameliorates DSS–induced colonic inflammation in a mouse colitis model through inhibition of the IL–6/STAT3 pathway. Mol Med Rep.

[23] Gou H, Su H, Liu D, Wong CC, Shang H, Fang Y, Zeng X, Chen H, Li Y, Huang Z, Fan M, Wei C, Wang X, Zhang X, Li X. and J. Yu.Traditional Medicine Pien Tze Huang Suppresses Colorectal Tumorigenesis through Restoring Gut Microbiota and Metabolites.Gastroenterology.2023. 10.1053/j.gastro.2023.08.052 37704113

[24] Shen A, Chen Y, Liu L, Huang Y, Chen H, Qi F, Lin J, Shen Z, Wu X, Wu M, Li Q, Qiu L, Yu N, Sferra TJ (2019). Peng.EBF1-Mediated upregulation of Ribosome Assembly factor PNO1 contributes to Cancer Progression by negatively regulating the p53 signaling pathway. Cancer Res.

[25] Kanehisa M, Furumichi M, Sato Y, Ishiguro-Watanabe M (2021). Tanabe.KEGG: integrating viruses and cellular organisms. Nucleic Acids Res.

[26] Sebio A, Kahn M (2014). Lenz.The potential of targeting Wnt/β-catenin in colon cancer. Expert Opin Ther Targets.

[27] Terzić J, Grivennikov S, Karin E. and M. Karin Inflamm colon Cancer Gastroenterol2010;138. 2101–2114.e5. 10.1053/j.gastro.2010.01.058 20420949

[28] Rothemich A. and J.C. Arthur.The Azoxymethane/Il10 (-/-) Model of Colitis-Associated Cancer (CAC).Methods Mol Biol.2019;1960. 215–225. 10.1007/978-1-4939-9167-9_19 30798535

[29] Kobayashi K, Tomita H, Shimizu M, Tanaka T, Suzui N, Miyazaki T. and A. Hara.p53 expression as a diagnostic biomarker in Ulcerative Colitis-Associated Cancer. Int J Mol Sci.2017;18. 10.3390/ijms18061284 PMC5486106 28621756

[30] Tan G, Huang C, Chen J. and F. Zhi.HMGB1 released from GSDME-mediated pyroptotic epithelial cells participates in the tumorigenesis of colitis-associated colorectal cancer through the ERK1/2 pathway.J Hematol Oncol.2020;13. 149. 10.1186/s13045-020-00985-0 PMC7648939 33160389

[31] Zeng S, Chen L, Sun Q, Zhao H, Yang H, Ren S, Liu M, Meng X (2021). Xu.Scutellarin ameliorates colitis-associated colorectal cancer by suppressing Wnt/β-catenin signaling cascade. Eur J Pharmacol.

[32] Jiang F, Liu M, Wang H, Shi G, Chen B, Chen T, Yuan X, Zhu P, Zhou J, Wang Q. and Y. Chen.Wu Mei Wan attenuates CAC by regulating gut microbiota and the NF-kB/IL6-STAT3 signaling pathway.Biomed Pharmacother.2020;125. 109982. 10.1016/j.biopha.2020.109982 32119646

[33] Shenoy AK, Fisher RC, Butterworth EA, Pi L, Chang LJ, Appelman HD, Chang M, Scott EW. and E.H. Huang.Transition from colitis to cancer: high wnt activity sustains the tumor-initiating potential of colon cancer stem cell precursors. Cancer Res.2012;72. 5091 – 100. 10.1158/0008-5472.CAN-12-1806 PMC3463774 22902411

[34] Reya T. and H. Clevers.Wnt signalling in stem cells and cancer.Nature.2005;434. 843 – 50. 10.1038/nature03319 15829953

[35] Koch S (2017). Extrinsic control of wnt signaling in the intestine. Differentiation.

